# Functional gene polymorphisms and expression alteration of selected microRNAs and the risk of various gastric lesions in *Helicobacter pylori*-related gastric diseases

**DOI:** 10.3389/fgene.2022.1097543

**Published:** 2023-01-12

**Authors:** Qi Liu, Danyan Li, Yunkai Dai, Yunzhan Zhang, Shaoyang Lan, Qi Luo, Jintong Ye, Xu Chen, Peiwu Li, Weijing Chen, Ruliu Li, Ling Hu

**Affiliations:** ^1^ Institute of Gastroenterology, Science and Technology Innovation Center, Guangzhou University of Chinese Medicine, Guangzhou, China; ^2^ Department of Gastroenterology, First Affiliated Hospital of Guangzhou University of Chinese Medicine, Guangzhou, China

**Keywords:** microRNA, *Helicobacter pylori*, gastric disease, single nucleotide polymorphisms, interaction

## Abstract

**Background:** *Helicobacter pylori* (Hp) persistent infection is an important pathogenic factor for a series of chronic gastric diseases from chronic gastritis to gastric cancer. Genetic and epigenetic abnormalities of microRNAs may play a vital role in the pathological evolution of gastric mucosa in *Helicobacter pylori*-related gastric diseases (HPGD). This study aimed to investigate the relationship between miR-146a, miR-196a2, miR-149, miR-499 and miR-27a gene single nucleotide polymorphisms (SNPs) and their expressions with pathological changes in gastric mucosa, and to further analyze the interactions between SNPs and Hp.

**Methods:** Subjects in this study included patients diagnosed with HPGD and healthy controls. MiR-146a rs2910164, miR-196a2 rs11614913, miR-149 rs2292832, miR-499 rs3746444 and miR-27a rs895819 were genotyped by direct sequencing. Fluorescence quantitative PCR was used to detect microRNA expressions. Gene-gene and gene-environment interactions were evaluated by multifactor dimensionality reduction (MDR) method.

**Results:** we found that frequency distribution of miR-196a2 rs11614913 CT genotype in gastric precancerous lesion (GPL) group and gastric cancer (GC) group was significantly higher than normal control (NOR) group [adjusted OR = 6.16, 95%CI (1.46–26.03); adjusted OR = 11.83, 95%CI (1.65–84.72), respectively]. CT genotype and C allele of miR-27a rs895819 were associated with increased risk of GC [adjusted OR = 10.14, 95%CI (2.25–45.77); adjusted OR = 3.71, 95%CI(1.46–9.44), respectively]. The MDR analysis results showed that the interaction between miR-196a2 rs11614913 and Hp was associated with the risk of GPL (*p* = 0.004). Meanwhile, the expression level of miR-196a2 in GC group was significantly higher than NOR, chronic inflammation (CI) and early precancerous lesion (EPL) groups among Hp-positive subjects. And expressions of miR-499 and miR-27a in GC group were both higher than EPL group. Also, miR-27a expression in GC group was higher than CI and gastric atrophy (GA) groups.

**Conclusion:** miR-196a2 rs11614913 and miR-27a rs895819 may affect the genetic susceptibility to GPL or GC. MiR-196a2 rs11614913 and Hp have a synergistic effect in the occurrence and development of GPL. The up-regulation of miR-499, miR-196a2 and miR-27a expression caused by Hp infection may be an important mechanism of gastric carcinogenesis.

## Introduction

*Helicobacter pylori*-related gastric diseases (HPGD) are a group of digestive system diseases induced by Hp infection, such as chronic superficial gastritis, gastric ulcer, atrophic gastritis and gastric cancer (Kamangar et al., 2011; Bravo et al., 2018). They cover the entire pathological evolution of gastric mucosa from chronic inflammation to atrophy, intestinal metaplasia, dysplasia and carcinogenesis. As the main pathogenic factor of HPGD, Hp selectively colonizes in approximately half of human gastric mucosa worldwide (Venneman et al., 2018). However, the clinical outcomes of Hp infection are not influenced by bacterial virulence factors alone. The role played by host genetic susceptibility factors is also becoming more apparent (Mohammadi et al., 2022).

MicroRNAs (miRNAs) are a class of non-coding RNAs encoded by endogenous genes and are approximately 22 nucleotides in length. They can specifically bind to the 3′untranslated region of target gene mRNAs, triggering mRNA degradation and regulating genes (Zlotorynski, 2019). Functionally, miRNAs are involved in biological processes like signal transduction, response to host infection, development and differentiation, and are closely linked to the occurrence and progression of cancer (Bartel, 2004). Studies have reported that single nucleotide polymorphisms (SNPs) in coding sequence of precursor miRNA genes may influence the levels of mature miRNA, thereby altering the individual’s susceptibility to related diseases (Nicoloso et al., 2010). In addition, the importance of gene-gene and gene-environment interactions in the development of cancer and its susceptibility cannot be overlooked. Kim et al. (2016) proposed that more than two genes may indirectly interact with each other and establish a topological network in affecting gastrointestinal cancer susceptibility. MiR-146a, miR-196a2, miR-149, miR-499 and miR-27a are involved in the pathogenesis of various cancers, including breast, colorectal, hepatocellular, and gastric cancers (Peng et al., 2010; Min et al., 2012; Bansal et al., 2014; Chen et al., 2016). These miRNA-related SNPs (rs2910164, rs11614913, rs2292832, rs3746444, rs895819) can affect their functions by changing the efficiency of miRNA maturation, expression and/or targeting (Xu et al., 2017; Zou et al., 2018). However, few studies have explored the associations between these five miRNAs and different pathological alterations in gastric mucosa of Chinese HPGD population and their possible synergistic effects with Hp infection.

Our work sought to investigate the associations of miR-146a, miR-149, miR-196a2, miR-27a and miR-499 gene polymorphisms and their expressions with different pathological alterations in HPGD subjects’ gastric mucosa. And we also aimed to further explore the potential interactions between SNPs, and between SNPs and Hp infection in gastric precancerous lesions and gastric cancer susceptibility.

## Materials and methods

### Study population

This study was a clinical retrospective study. A total of 203 participants were recruited from the endoscopy center and gastrointestinal surgery of the first affiliated hospital of Guangzhou University of Chinese Medicine. All participants consisted of healthy people who underwent routine physical examination and patients diagnosed with chronic superficial gastritis, gastric ulcer, atrophic gastritis or gastric cancer by endoscopy and histopathological examination. The criteria for performing endoscopy referred to the ASGE guideline (Early et al., 2012). Subjects with a history of other serious systemic diseases and other malignancies were excluded. The healthy control group in this study included individuals with normal gastric mucosa or only mild chronic inflammation without other lesions. Gastric mucosa specimens of each subject were taken for subsequent Hp infection, histopathological changes of gastric mucosa, miRNA gene polymorphism and expression level detection. The study protocol was approved by the Ethics Committee of the First Affiliated Hospital of Guangzhou University of Chinese Medicine [ethics approval No. (2015) 009]. All subjects signed the informed consent.

### Inclusion and exclusion criteria

The detailed inclusion and exclusion criteria of patients were as follow: Inclusion criteria: 1) Those who meet the diagnostic criteria for those above gastric diseases; 2) Age between 18 and 80 years old; 3) Those who voluntarily participated in the study and signed the informed consent form. Exclusion criteria: 1) Patients combined with other serious systemic diseases; 2) Those who took traditional Chinese medicine, PPI or Hp eradication treatment in the past month; 3) Pregnant and breastfeeding women; 4) Those with a history of severe mental illness or intellectual disability who are unable to cooperate with the collection of medical history data and sign the informed consent form.

### Genotyping

HiPure tissue DNA kit (Magen, Guangzhou, China) was used to extract genomic DNA from gastric mucosal tissues. Polymerase chain reaction was performed by PCR DSMIX kit (Dongsheng Biotech, Guangdong, China). The primer sequences of the five miRNAs were as follow: 5′-CGA​CTC​TCT​ATG​AGA​ATT​ATG​C-3′ (forward) and 5′-AAT​TGG​ATG​CCG​CAG​TGG​TC-3′ (reverse) for miR-499; 5′-TCCAGATAGATGCAAAGCTG-3′ (forward) and 5′-AGA​CTC​CTCTC​CCT​TAA​TCA​C-3′ (reverse) for miR-196a2; 5′-CAT​CTC​ATG​TCC​AGG​ACC​AC-3′ (forward) and 5′-AGTGAGCTGGTCCAAGACTCAG-3′ (reverse) for miR-149; 5′-GAC​CTG​GTA​CTA​GGA​AGC​AG-3′ (forward) and 5′-CTT​ATA​CCT​TCA​GAC​CTG​AG-3′ (reverse) for miR-146; 5′-TGT​GTT​TCA​GCT​CAG​TAG​GCA​C-3′ (forward) and 5′-CTG​TCA​CAA​ATC​ACA​TTGCC-3′ (reverse) for miR-27a. The PCR reaction procedure was as follows: for miR-499 and miR-149, pre-deformation at 94°C for 4 min, followed by denaturation at 94°C for 30 s, annealing at 60°C for 30 s, and 72°C for 30 s for a total of 40 cycles; finally, extension at 72°C for 10 min and storage at 10°C for 30 s. For miR-196a2 and miR-146a, pre-deformation at 94°C for 4 min, followed by denaturation at 94°C for 30 s, annealing at 60°C for 30 s, and 72°C for 30 s for a total of 32 cycles; finally, extension at 72°C for 10 min and storage at 10°C for 30 s. For miR-27a, pre-deformation at 94°C for 4 min, followed by denaturation at 94°C for 30 s, annealing at 60°C for 30 s, and 72°C for 30 s for a total of 35 cycles; finally, extension at 72°C for 10 min and storage at 10°C for 30 s. The PCR products were identified by 1% agarose gel electrophoresis. After purification, PCR products were cycle sequenced using BigDye Terminator v3.1 cycle Sequencing kit following ABI standard procedures.

### Fluorescent quantitative PCR

Total RNA extraction was performed using Hipure Universal miRNA Kit (Magen). The isolated RNA was reverse transcribed to cDNA using the ToYoBo qPCR RT Kit (ToYoBo). KAPA Probe Fast qPCR Master Mix (KAPA BIOSYSTEMS) was used for fluorescent qPCR reactions. The expression level of U6 gene was used as the internal reference for target miRNA genes. The primer sequences of target genes and U6 were shown in Supplementary Table S1. The PCR reaction conditions were: 95°C for 3 min, followed by 40 cycles (95°C for 3 s, 55°C for 30 s) and 30°C for 30 s. Three replicate measurements were performed for each sample. 2-ΔΔCq method was used to calculate the expressions of miRNAs (Livak and Schmittgen, 2001).

### Hp detection

The Kyoto consensus (Sugano et al., 2015) as well as the New Sydney system (Dixon et al., 1996) were consulted for the diagnostic criteria of Hp infection. Rapid urokinase test and methylene blue staining were applied to detect Hp infection. As long as one of these tests was positive, the subject was considered to be Hp-infected. According to the distribution of Hp, the degree of Hp infection was classified into four levels: negative, mild, moderate and severe.

### Gastric mucosal histopathological criteria and grouping

The histopathological changes of gastric mucosa were observed by hematoxylin-eosin staining. All slides were reviewed blindly by two trained pathologists. According to the new Sydney system (Dixon et al., 1996), the severity of inflammation, inflammatory activity, atrophy, intestinal metaplasia and dysplasia in subjects’ gastric mucosa was divided into four grades: none, mild, moderate, and severe. Based on the above results and research needs, we divided all subjects into five different pathological groups as follows: 1) Normal control group (NOR): those with basically normal gastric mucosa or only with mild inflammation; 2) Chronic inflammation group (CI): Those with moderate to severe inflammation in gastric mucosa; 3) Gastric atrophy group (GA): Those whose gastric mucosa with glandular atrophy as the main manifestation, not accompanied by intestinal metaplasia as well as dysplasia; 4) Gastric precancerous lesion group (GPL): Gastric mucosa atrophy with intestinal metaplasia or/and dysplasia; 5) Gastric cancer group (GC): Patients diagnosed with gastric cancer. Considering that severe dysplasia is more likely to become cancerous in precancerous lesions of gastric cancer, the gastric precancerous lesion group (GPL) was subdivided into two subgroups: early precancerous lesion group (EPL) and severe dysplasia group (SE). Early precancerous lesion group (EPL): Those with gastric atrophy accompanied by intestinal metaplasia and/or mild to moderate dysplasia; Severe dysplasia group (SE): those with severe dysplasia in gastric mucosa.

### Statistical analysis

Based on normality of data, continuous variables were expressed as mean ± standard deviation or median (first quartile, third quartile). Categorical variables were analyzed by χ^2^ test or Fisher exact test. Kruskal-Wallis H test was used to estimate the differences in miRNA expression levels between various groups. Hardy-Weinberg equilibrium test of SNPs was performed using χ^2^ test or Fisher exact test. Binary logistic regression was used to evaluate the odds ratio (OR) and 95% confidence interval (CI) for the associations between miRNA gene polymorphism and GPL as well as GC risk. Spearman correlation analysis was used to examine the correlations between miRNA expressions and severity of Hp infection as well as histopathological changes of gastric mucosa. Gene-gene and gene-environment interactions were explored by multifactor dimensionality reduction (MDR) method. 1000 permutation test was used to assess the statistical significance of MDR interaction models. The above data analysis was carried out using SPSS 21, GraphPad Prism 8.0, open source multifactor dimensionality reduction (MDR) software (V3.0.2), and MDR permutation test software BETA version 0.4.6. *p* < 0.05 was considered statistically significant.

## Results

### Study population

**FIGURE 1:**
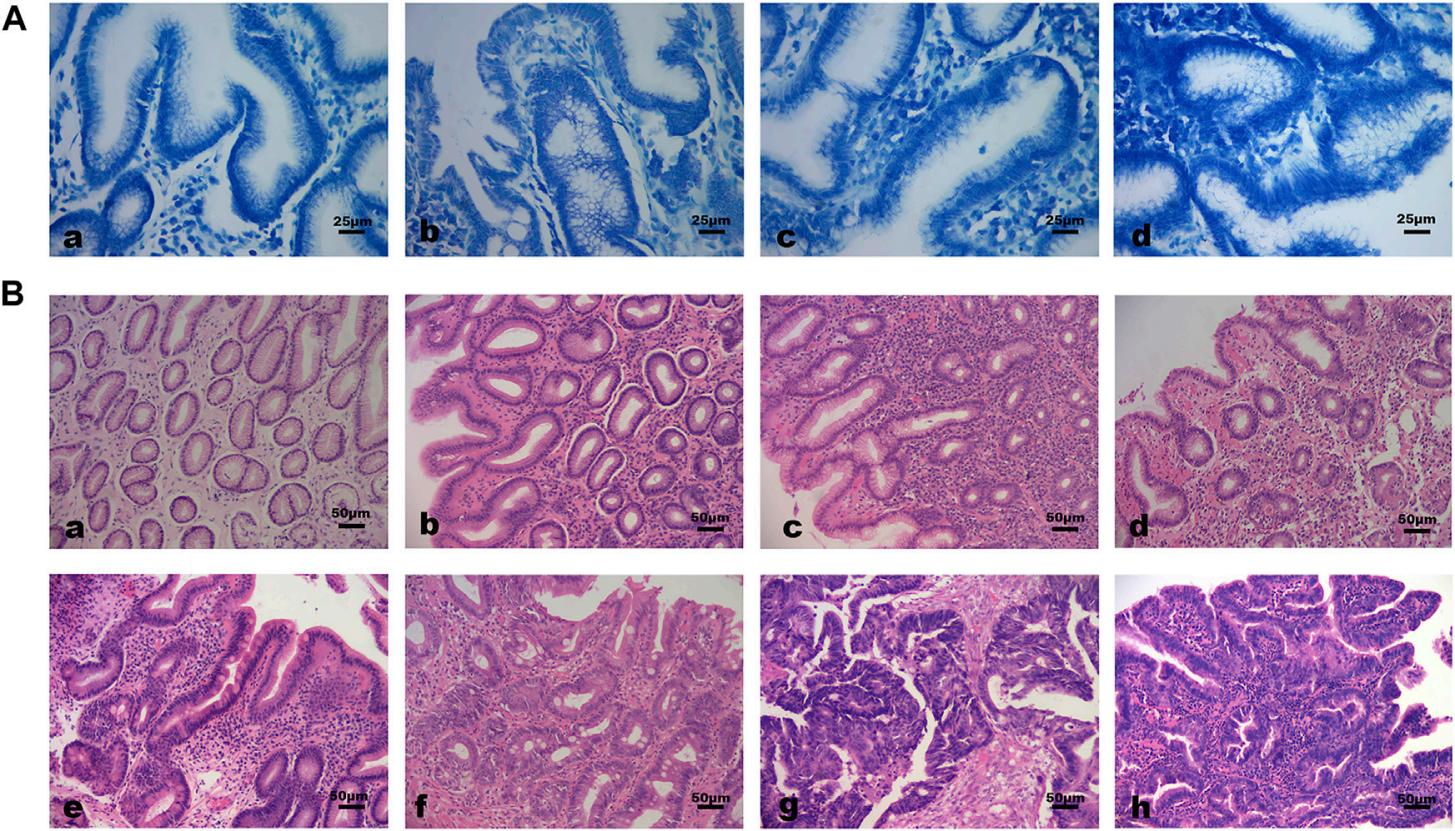
Different Hp infection states and histopathological features of gastric mucosa. **(A)** Hp infection situations (methylene blue staining 400×): **(a)** Negative; **(b)** Mild; **(c)** Moderate; **(d)** Severe. **(B)** Histopathological features of gastric mucosa (hematoxylin-eosin staining 200×): **(a)** Normal gastric mucosa; **(b)** Gastric mucosa with moderate inflammation; **(c)** Gastric mucosa with moderate atrophy and severe inflammation; **(d)** Moderate atrophic gastric mucosa with mild dysplasia and moderate inflammation; **(e)** Moderate atrophic gastric mucosa with mild intestinal metaplasia, moderate dysplasia and severe inflammation; **(f)** Gastric mucosa with severe dysplasia and moderate intestinal metaplasia. **(g, h)** Cancerous gastric mucosa.

The baseline characteristics of subjects were shown in Table 1. The differences in the distribution of Hp infection severity between different pathological groups were statistically significant. The rate of Hp infection was significantly higher in GPL and GC group than in NOR group (all *p* < 0.01). Specifically, there were more moderate to severe Hp infected subjects in GPL group than in CI and NOR group (*p* = 0.001, *p* < 0.001). There was no significant difference in age and sex distribution among pathological groups. Figure 1 demonstrated different Hp infection situations and histopathological features in gastric mucosa of subjects.

**TABLE 1:** Demographic information of study subjects.

Variables	NOR (%) (*n* = 35)	CI(%) (*n* = 23)	GA (%) (*n* = 11)	GPL (%) (*n* = 89)	GC (%) (*n* = 45)	*P* ^a^ ^,^ ^b^ ^,^ ^c^
Age (Mean ± SD)	47.11 ± 12.02	48.22 ± 14.62	49.91 ± 18.90	48.42 ± 11.06	54.09 ± 14.83	0.20^a^
Gender (male)	18 (51.43)	11 (47.83)	6 (54.55)	58 (65.17)	32 (71.11)	0.21^b^
*H. pylori*						<0.001^c^
negative	16 (45.71)	8 (34.78)	0 (0.00)	6 (6.74)	6 (13.33)	
mild	19 (54.29)	14 (60.87)	7 (63.64)	46 (51.69)	32 (71.11)	
moderate	0 (0.00)	1 (4.35)	1 (9.09)	13 (14.61)	6 (13.33)	
severe	0 (0.00)	0 (0.00)	3 (27.27)	24 (26.97)	1 (2.22)	

NOR, Normal control group; CI, Chronic inflammation group; GA, Gastric atrophy group; GPL, Gastric precancerous lesions group; GC, Gastric cancer group.

^a^ *p*-value was calculated by One-way analysis of variance (ANOVA).

^b^ *p*-value was calculated by chi-square test.

^c^ *p*-value was calculated by Fisher’s exact test.

### The genotyping results of miRNAs

**FIGURE 2:**
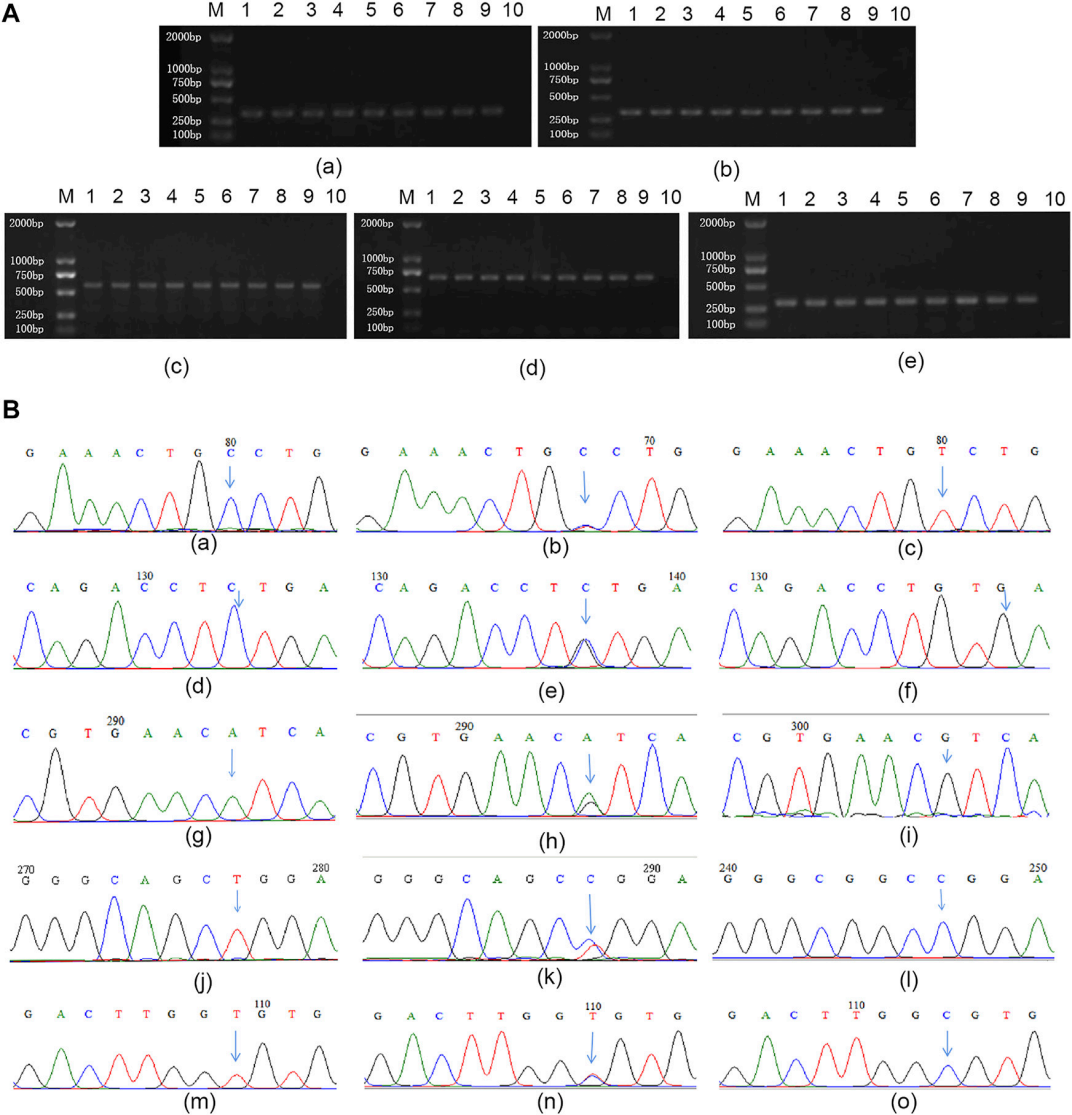
Genotyping for miRNA SNPs **(A)** representative images of gel electrophoresis of PCR products: **(a–e)** represent miR-196a2, miR-146a, miR-499, miR-149, and miR-27a respectively. M: Marker; 1 and 10 are positive and negative control, respectively; Lane 2 to 9 represent 8 different individuals **(B)** Results of genotyping. **(a–c)**: CC homozygous wild, CT heterozygous mutant and TT homozygous mutant type of rs11614913; **(d–f)**: CC homozygous wild, CG heterozygous mutant and GG homozygous mutant type of rs2910164; **(g–i)**: AA homozygous wild, AG heterozygous mutant and GG homozygous mutant type of rs3746444; **(j–l)**: TT homozygous wild, CT heterozygous mutant and CC homozygous mutant type of rs2292832; **(m–o)**: TT homozygous wild, CT heterozygous mutant and CC homozygous mutant type of rs895819.

Different genotypes of all miRNA SNPs were detected by PCR direct sequencing, and the results were shown in Figure 2.

### Genotype and allele frequencies of miRNA polymorphisms in GPL and GC subjects

The results of Hardy Weinberg equilibrium test showed that the distribution of different genotypes in GPL, GC and NOR groups were in Hardy Weinberg equilibrium (all *p* > 0.05) (Table 2). The frequencies of different genotypes and alleles of miR-499 rs3746444, miR-149 rs2292832, miR-196a2 rs11614913, miR-146a rs291016 and miR-27a rs895819 in GPL, GC group and NOR group were presented in Table 2. The frequency of CT genotype at miR-196a2 rs11614913 locus was significantly higher in GPL and GC group than in NOR group [CT *vs*. CC: adjusted OR = 6.16, 95%CI (1.46–26.03), *p* = 0.01; adjusted OR = 11.83, 95%CI (1.65–84.72), *p* = 0.01, respectively]. In addition, the frequency distribution of CT genotype and C allele of miR-27a rs895819 in GC group was significantly higher than in NOR group [CT *vs*. TT: adjusted OR = 10.14, 95%CI (2.25–45.77), *p* < 0.01; C *vs*. T: adjusted OR = 3.71, 95%CI (1.46–9.44), *p* = 0.01]. And the differences in genotypes and alleles distribution of the other three SNPs between GPL, GC group and NOR group were not statistically significant.

**TABLE 2:** Genotype and allele frequencies of miRNA gene polymorphisms in GPL, GC and control subjects.

SNPs	Genotype/Allele	NOR (%) (*n* = 35)	GPL (%) (*n* = 89)	GC (%) (*n* = 45)	*P*, Crude OR (95%CI)^a^	*P*, AOR (95%CI)^a^	*P*, Crude OR (95%CI)^b^	P, AOR (95%CI)^b^
miR-499 rs3746444	AA	28 (80.00)	70 (78.65)	35 (77.78)	1.00 (reference)	1.00 (reference)	1.00 (reference)	1.00 (reference)
	AG	7 (20.00)	18 (20.22)	9 (20.00)	0.50, 0.69 (0.24–2.00)	0.62, 0.73 (0.21–2.53)	0.63, 0.72 (0.19–2.69)	0.92, 0.92 (0.19–4.45)
	GG	0 (0.00)	1 (1.12)	1 (2.22)	-	-	-	-
	A	63 (90.00)	158 (88.76)	79 (87.78)	1.00 (reference)	1.00 (reference)	1.00 (reference)	1.00 (reference)
	G	7 (10.00)	20 (11.24)	11 (12.22)	0.87, 0.92 (0.35–2.41)	0.85, 0.90 (0.31–2.65)	0.84, 1.11 (0.39–3.18)	0.62, 1.34 (0.42–4.26)
	*HWE* P	-	1.00	1.00				
miR-149 rs2292832	TT	15 (42.86)	36 (40.45)	21 (46.67)	1.00 (reference)	1.00 (reference)	1.00 (reference)	1.00 (reference)
	CT	17 (48.57)	48 (53.93)	17 (37.78)	0.57, 1.29 (0.53–3.15)	0.33, 1.67 (0.59–4.72)	0.35, 0.59 (0.20–1.78)	0.33, 0.51 (0.13–1.99)
	CC	3 (8.57)	5 (5.62)	7 (15.56)	0.61, 0.66 (0.13–3.36)	0.41, 0.45 (0.07–2.99)	0.57, 1.63 (0.30–9.04)	0.95, 0.93 (0.12–7.55)
	T	47 (67.14)	120 (67.42)	59 (65.56)	1.00 (reference)	1.00 (reference)	1.00 (reference)	1.00 (reference)
	C	23 (32.86)	58 (32.58)	31 (34.44)	0.97, 1.00 (0.52–1.89)	0.91, 1.04 (0.49–2.20)	0.67, 1.17 (0.57–2.42)	0.99, 1.00 (0.44–2.29)
	*HWE* P	0.89	0.32	0.72				
miR-196a2 rs11614913	CC	12 (34.29)	10 (11.24)	6 (13.33)	1.00 (reference)	1.00 (reference)	1.00 (reference)	1.00 (reference)
	CT	13 (37.14)	53 (59.55)	24 (53.33)	<0.01, 6.78 (2.15–21.45)	<0.01, 7.31 (1.86–28.81)	0.01, 6.16 (1.46–26.03)	0.01, 11.83 (1.65–84.72)
	TT	10 (28.57)	26 (29.21)	15 (33.33)	0.03, 3.82 (1.15–12.69)	0.03, 4.92 (1.18–20.47)	0.10, 3,21 (0.79–13.09)	0.07, 4.96 (0.88–27.93)
	C	37 (52.86)	73 (41.01)	36 (40.00)	1.00 (reference)	1.00 (reference)	1.00 (reference)	1.00 (reference)
	T	33 (47.14)	105 (58.99)	54 (60.00)	0.06, 1.84 (0.98–3.45)	0.06, 1.98 (0.96–4.07)	0.07, 1.92 (0.94–3.91)	0.13, 1.85 (0.83–4.14)
	*HWE* P	0.63	0.29	0.91				
miR-146a rs2910164	CC	14 (40.00)	31 (34.83)	15 (33.33)	1.00 (reference)	1.00 (reference)	1.00 (reference)	1.00 (reference)
	CG	16 (45.71)	48 (53.93)	24 (53.33)	0.74, 1.17 (0.47–2.92)	0.44, 0.65 (0.22–1.94)	0.65, 1.29 (0.43–3.87)	0.88, 1.11 (0.30–4.15)
	GG	5 (14.29)	10 (11.24)	6 (13.33)	0.38, 0.54 (0.13–2.17)	0.50, 0.56 (0.11–2.97)	0.88, 1.13 (0.24–5.40)	0.39, 2.26 (0.36–14.33)
	C	44 (62.86)	110 (61.80)	54 (60.00)	1.00 (reference)	1.00 (reference)	1.00 (reference)	1.00 (reference)
	G	26 (37.14)	68 (38.20)	36 (40.00)	0.75, 0.90 (0.48–1.70)	0.50, 0.78 (0.38–1.60)	0.81, 1.09 (0.54–2.23)	0.40, 1.42 (0.63–3.21)
	*HWE* P	1.00	0.63	0.91				
miR-27a rs895819	TT	22 (62.86)	50 (56.18)	19 (42.22)	1.00 (reference)	1.00 (reference)	1.00 (reference)	1.00 (reference)
	CT	11 (31.43)	31 (34.83)	23 (51.11)	0.38, 1.53 (0.60–3.89)	0.22, 2.05 (0.65–6.47)	0.03, 3.36 (1.15–9.85)	<0.01, 10.14 (2.25–45.77)
	CC	2 (5.71)	8 (8.99)	3 (6.67)	0.22, 3.34 (0.48–22.97)	0.60, 1.84 (0.19–17.65)	0.43, 2.49 (0.26–23.55)	0.11, 7.91 (0.62–100.80)
	T	55 (78.57)	131 (73.60)	61 (67.78)	1.00 (reference)	1.00 (reference)	1.00 (reference)	1.00 (reference)
	C	15 (21.43)	47 (26.40)	29 (32.22)	0.24, 1.53 (0.76–3.10)	0.34, 1.49 (0.66–3.36)	0.07, 2.03 (0.94–4.39)	0.01, 3.71 (1.46–9.44)
	*HWE* P	1.00	0.75	0.67				

NOR, Normal control group; GPL, Gastric precancerous lesions group; GC, Gastric cancer group; HWE, Hardy-Weinberg equilibrium; OR, Odds ratio; CI, Confidence interval; AOR, Adjusted odds ratio, adjusted for age, gender and *H. pylori* infection.

^a^ GPL *vs*. NOR.

^b^ GC *vs*. NOR.

### Combined allele frequencies of miRNA polymorphisms in GPL and GC subjects

We analyzed the frequency distribution of allele combinations at the five miRNA (miR-499/miR-149/miR-196a2/miR-146a/miR-27a) SNPs in GPL, GC group and NOR group to assess the potential synergistic effects among miRNAs. Compared with NOR group, the frequency of A-T-C-C-T allele combination was significantly lower and the frequencies of A-T-T-G-C and A-C-T-C-T allele combination were significantly higher in GC group (all *p* < 0.05) (Table 3). While in GPL group, the frequencies of A-T-C-G-T, A-T-T-G-T, G-T-T-C-T and G-T-T-G-T allele combinations were significantly lower and the frequencies of A-C-C-C-T, A-C-C-C-C, A-C-T-C-T and A-C-T-C-C allele combinations were significantly higher (all *p* < 0.05) (Table 3).

**TABLE 3:** Frequencies of allele combinations of miRNA gene polymorphisms in GPL, GC and control subjects.

Allele combination	NOR (%) (2n = 70)	GPL (%) (2n = 178)	GC (%) (2n = 90)	*P,* OR (95%CI)^a^	*P,* OR (95%CI)^b^
miR-499/miR-149/miR-196a2/miR-146a/miR-27a					
A-T-C-C-T	12 (17.14)	31 (17.42)	2 (2.22)	0.96, 1.02 (0.49–2.12)	<0.01, 0.11 (0.02–0.51)
A-T-C-C-C	2 (2.86)	16 (8.99)	0 (0.00)	0.10, 3.36 (0.75–15.01)	0.19, 0.97 (0.93–1.01)
A-T-C-G-T	6 (8.57)	3 (1.69)	6 (6.67)	0.02, 0.18 (0.04–0.75)	0.65, 0.76 (0.24–2.47)
A-T-C-G-C	0 (0.00)	3 (1.69)	0 (0.00)	0.56, 1.02 (1.00–1.04)	-
A-T-T-C-T	8 (11.43)	14 (7.87)	12 (13.33)	0.37, 0.66 (0.27–1.65)	0.72, 1.19 (0.46–3.10)
A-T-T-C-C	0 (0.00)	6 (3.37)	0 (0.00)	0.19, 1.04 (1.01–1.06)	-
A-T-T-G-T	24 (34.29)	6 (3.37)	31 (34.44)	<0.001, 0.07 (0.03–0.17)	0.98, 1.01 (0.52–1.94)
A-T-T-G-C	0 (0.00)	3 (1.69)	7 (7.78)	0.56, 1.02 (1.00–1.04)	0.02, 1.08 (1.02–1.15)
A-C-C-C-T	2 (2.86)	24 (13.48)	2 (2.22)	0.01, 5.30 (1.22–23.06)	1.00, 0.77 (0.11–5.63)
A-C-C-C-C	0 (0.00)	24 (13.48)	0 (0.00)	<0.01, 1.16 (1.09–1.23)	-
A-C-C-G-T	0 (0.00)	2 (1.12)	2 (2.22)	1.00, 1.01 (1.00–1.03)	0.51, 1.02 (1.00–1.06)
A-C-C-G-C	0 (0.00)	5 (2.81)	0 (0.00)	0.33, 1.03 (1.00–1.06)	-
A-C-T-C-T	0 (0.00)	17 (9.55)	6 (6.67)	<0.01, 1.12 (1.05–1.16)	0.04, 1.07 (1.01–1.13)
A-C-T-C-C	0 (0.00)	15 (8.43)	0 (0.00)	0.01, 1.09 (1.04–1.14)	-
A-C-T-G-T	2 (2.86)	1 (0.56)	2 (2.22)	0.19, 0.19 (0.02–2.15)	1.00, 0.77 (0.12–5.63)
A-C-T-G-C	0 (0.00)	4 (2.25)	0 (0.00)	0.58, 1.02 (1.00–1.05)	-
G-T-C-C-C	0 (0.00)	2 (1.12)	0 (0.00)	1.00, 1.01 (1.00–1.03)	-
G-T-C-G-C	2 (2.86)	0 (0.00)	0 (0.00)	0.08, 0.97 (0.93–1.01)	0.19, 0.97 (0.93–1.01)
G-T-T-C-T	4 (5.71)	1 (0.56)	8 (8.89)	0.02, 0.09 (0.01–0.85)	0.33, 1.83 (0.53–6.37)
G-T-T-G-T	6 (8.57)	1 (0.56)	8 (8.89)	<0.01, 0.06 (0.01–0.51)	0.94, 1.04 (0.34–3.15)
G-T-T-G-C	0 (0.00)	0 (0.00)	2 (2.22)	-	0.51, 1.02 (0.99–1.06)
G-C-T-G-T	2 (2.86)	0 (0.00)	2 (2.22)	0.08, 0.97 (0.93–1.01)	1.00, 0.77 (0.11–5.63)

NOR, Normal control group; GPL, Gastric precancerous lesions group; GC, Gastric cancer group.

^a^ GPL *vs*. NOR.

^b^ GC *vs*. NOR.

### Gene-gene and gene-environment interactions analysis

The MDR method was used to explore whether there were gene-gene and gene-environment interactions between miRNA SNPs and Hp. As illustrated in Table 4, the second-order interaction model of rs3746444-rs11614913 was the best model with highest testing balanced accuracy of 56.4% among gene-gene interaction models for GPL susceptibility (Table 4). And within the gene-gene interaction models for GC susceptibility, the third-order interaction model of rs2292832-rs11614913-rs2910164 had the largest testing balanced accuracy of 61.1% and cross-validation consistency of 100% (Table 4), which was the best model. However, neither of the above two best models was statistically significant based on 1000 permutation tests. According to the results of gene-environment interaction analysis, only the second-order interaction model of rs11614913-Hp was statistically significant in two-order and higher-order models for GPL susceptibility (Table 5).

**TABLE 4:** Gene-gene interaction analysis for GPL and GC susceptibility.

Models	Training bal Acc	Testing bal Acc	CV consistency	P*
Model 1				
rs11614913	0.622	0.540	10/10	0.274
rs3746444, rs11614913	0.652	0.564	7/10	0.214
rs2292832, rs11614913, rs2910164	0.706	0.484	6/10	0.601
rs2292832, rs895819, rs11614913, rs2910164	0.751	0.392	7/10	0.951
rs3746444, rs2292832, rs895819, rs11614913, rs2910164	0.782	0.460	10/10	0.725
Model 2				
rs895819	0.616	0.481	7/10	0.553
rs895819, rs11614913	0.665	0.519	5/10	0.407
rs2292832, rs11614913, rs2910164	0.774	0.611	10/10	0.088
rs2292832, rs895819, rs11614913, rs2910164	0.830	0.559	10/10	0.218
rs3746444, rs2292832, rs895819, rs11614913, rs2910164	0.858	0.516	10/10	0.409

GPL, Gastric precancerous lesions group; GC, Gastric cancer group; Bal Acc, Balanced accuracy; CV, Cross-validation.

Model 1, Gene-gene interaction models for GPL susceptibility; Model 2, Gene-gene interaction models for GC susceptibility.

*p-values were based on 1000 permutation testing.

**TABLE 5:** Gene-environment interaction analysis for GPL and GC susceptibility.

Models	Training bal Acc	Testing bal Acc	CV consistency	P*
Model 1				
Hp	0.695	0.695	10/10	<0.001
rs11614913, Hp	0.712	0.653	7/10	0.004
rs2292832, rs11614913, Hp	0.746	0.565	6/10	0.192
rs3746444, rs2292832, rs11614913, Hp	0.786	0.593	5/10	0.100
rs3746444, rs2292832, rs11614913, rs2910164, Hp	0.833	0.536	8/10	0.284
rs3746444, rs2292832, rs895819, rs11614913, rs2910164, Hp	0.879	0.479	10/10	0.622
Model 2				
Hp	0.662	0.662	10/10	0.001
rs895819, Hp	0.687	0.511	8/10	0.451
rs2292832, rs11614913, rs2910164	0.773	0.594	10/10	0.116
rs2292832, rs895819, rs11614913, rs2910164	0.831	0.540	8/10	0.293
rs2292832, rs895819, rs11614913, rs2910164, Hp	0.879	0.459	9/10	0.721
rs3746444, rs2292832, rs895819, rs11614913, rs2910164, Hp	0.895	0.446	10/10	0.796

GPL, Gastric precancerous lesions group; GC, Gastric cancer group; Bal Acc, Balanced accuracy; CV, Cross-validation.

Model 1, Gene-environment interaction models for GPL susceptibility; Model 2, Gene-environment interaction models for GC susceptibility.

*p-values were based on 1000 permutation testing.

### Comparison of miRNAs’ expression levels in different pathological groups

**FIGURE 3:**
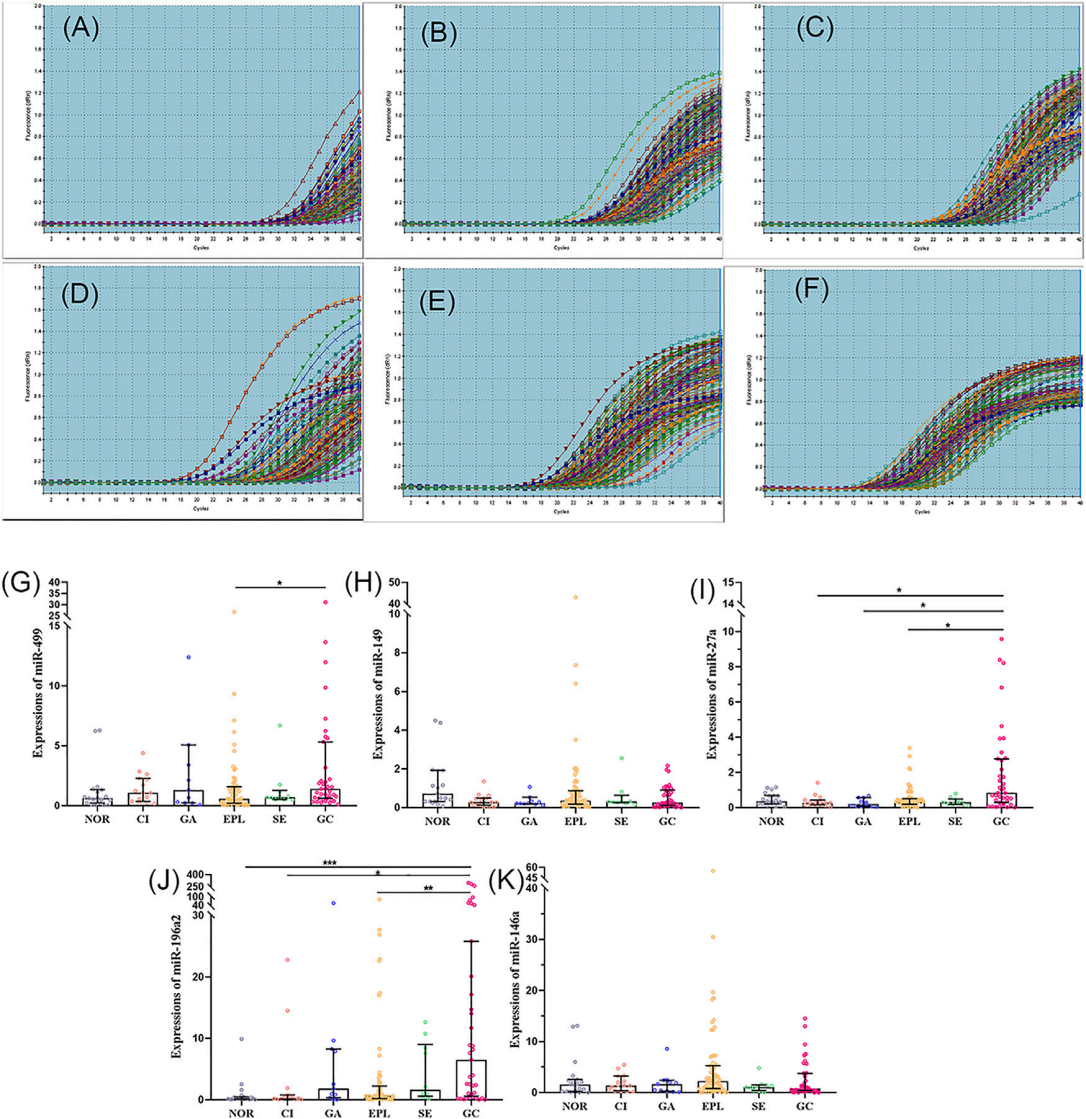
Comparison of miRNA expression levels among different pathological groups in Hp-positive subjects. **(A–F)**: amplification curves of miR-499, miR-149, miR-27a, miR-196a2, miR-146a and U6, respectively. **(G–K)**: comparison of miRNA expression levels of miR-499, miR-149, miR-27a, miR-196a2, and miR-146a between different pathological groups in Hp-positive subjects. The column height indicates the median expression level of miRNAs and the error bars indicate interquartile range. NOR, Normal control group; CI, Chronic inflammation group; GA, Gastric atrophy group; GPL, Gastric precancerous lesion group; EPL, Early precancerous lesion group; SE, Severe dysplasia group; GC, Gastric cancer group. *p < 0.05; **p < 0.01; ***p < 0.001.

To investigate the effect of Hp infection on miRNA expression levels in each pathological group, we divided all subjects into two parts for discussion according to whether Hp was infected or not. As displayed in Figure 3, expression levels of miR-499, miR-196a2 and miR-27 were statistically significant in different pathological groups among Hp positive subjects (*p* = 0.031, *p* = 0.002, *p* < 0.001, respectively). In particular, expression level of miR-499 was significantly higher in GC group than in EPL group (*p* = 0.029). And expression level of miR-27a was significantly up-regulated in GC group compared to CI, GA and EPL groups (*p* = 0.017, *p* = 0.021, *p* = 0.011). What’s more, expression level of miR-196a2 was significantly higher in GC group than in NOR, CI and EPL groups (*p* < 0.001, *p* = 0.015, *p* = 0.002). However, there was no significant difference in miRNA expression levels between different pathological groups among Hp-negative subjects (Supplementary Figure S1).

### The influence of SNPs on miRNAs’ expression levels

The results were revealed in Supplementary Table S2. The CC genotype of miR-146a rs2910164 was associated with lower miRNA expression level than CG/GG genotype in NOR, CI and GC groups (*p* = 0.025, *p* = 0.013, *p* = 0.024).

### Correlations of miRNAs’ expression levels with severity of Hp infection and grade of histopathology in gastric mucosa

**FIGURE 4:**
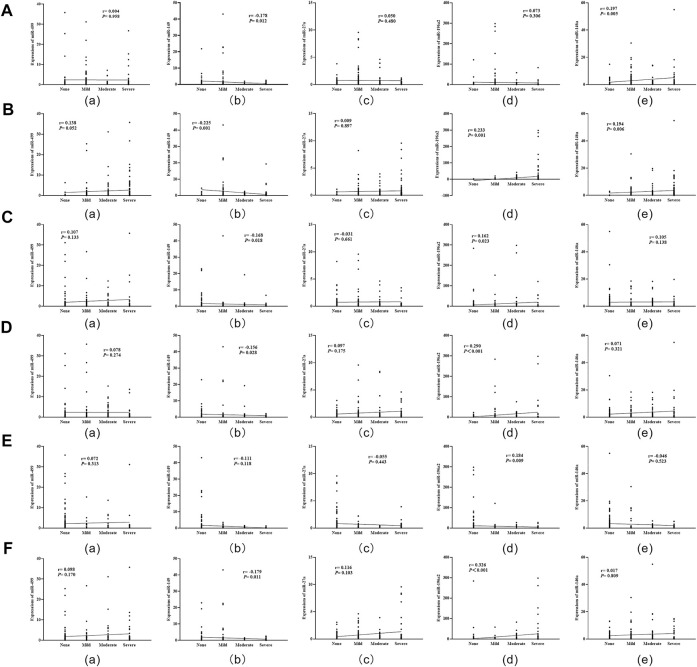
Correlation analysis of miRNA expression levels with severity of Hp infection and histopathology in gastric mucosa. **(A)**: Correlations of miRNA expression levels with Hp infection; **(B)**: Correlations of miRNA expression levels with degree of inflammation; **(C)**: Correlations of miRNA expression levels with inflammatory activity; **(D)**: Correlations of miRNA expression levels with degree of gastric atrophy; **(E)**: Correlations of miRNA expression levels with intestinal metaplasia; **(F)**: Correlations of miRNA expression levels with dysplasia.

Spearman correlation analysis showed that miR-196a2 expression level was positively correlated with the severity of inflammation, inflammatory activity, gastric atrophy, intestinal metaplasia and dysplasia (all *p* < 0.05) (Figure 4). Moreover, the expression level of miR-146a was positively correlated with degree of Hp infection and inflammation in gastric mucosa (all *p* < 0.05) (Figure 4). Conversely, miR-149 expression level were negatively correlated with severity of Hp infection, inflammation, inflammatory activity, atrophy and dysplasia (all *p* < 0.05) (Figure 4).

## Discussion

Hp is a gram-negative, spiral, micro-aerobic, flagellated bacteria that can selectively colonize the mucous layer of gastric mucosal epithelium. Interactions between host, gastric microenvironment and Hp bacterial virulence factors lead to chronic inflammation and further immune escape, and ultimately inducing malignant gastric mucosal lesions (Baj et al., 2021). MiRNAs are small molecule RNAs involving in the physiopathological processes of multiple diseases including cancer (Bartel, 2004). In present study, we explored the associations of miR-146a rs2910164, miR-149 rs2292832, miR-27a rs895819, miR-499 rs3746444 and miR-196a2 rs11614913 with GPL and GC risk. Besides, we further analyzed the influence of gene-gene and gene-environment interactions between these five SNPs and Hp infection on GPL and GC risk. Meanwhile, we also examined the links between miRNAs’ expressions and different pathological alterations in subjects’ gastric mucosa.

Our findings indicated that miR-196a2 rs11614913 was significantly associated with GPL and GC susceptibility. Compared with CC genotype, CT genotype carriers had an increased risk of GPL and GC. Similarly, Li et al. (2016) found an association between miR-196a2 rs11614913 and elevated GC risk. In addition, we identified that CT genotype and C allele of miR-27a rs895819 were associated with a higher risk of GC. A recent meta-analysis also demonstrated that miR-27a rs895819 may be associated with an increased risk of digestive system cancers (Yang et al., 2020). And we did not find any significant correlations between the remaining three miRNA SNPs (rs3746444, rs2292832, rs2910164) and GPL or GC risk. However, the associations between these five miRNA SNPs and GC susceptibility are still controversial. Xin et al. found no association between miR-196a2 rs11614913 polymorphism and GC risk in a northern Chinese population (Aziz et al., 2022). Another study reported that although there was no significant association between miR-27a rs895819 and gastrointestinal tumor risk, this SNP could synergize with other risk factors to increase the risk of gastrointestinal tumors (Shankaran et al., 2020). Meanwhile, several studies have found that there are different degrees of associations between miR-499 rs3746444, miR-149 rs2292832, miR-146a rs2910164 and GC risk (Zhang et al., 2018; Rong et al., 2021; Liang et al., 2022). And as we know, the occurrence and development of many diseases is a complex processes involving multifactor, multigene and multistage. While evaluating the accuracy of SNPs in predicting disease risk, it is important to assess the common influence of other susceptibility factors on the disease and the potential interactions between them (Schork et al., 2000). Therefore, we further investigated the interactions between these five miRNA SNPs and Hp infection in affecting the risk of GPL as well as GC. The results of combined allele analysis suggested that the frequencies distribution of many allele combinations were significantly higher or lower in GPL and GC groups compared with NOR group, indicating an association between GPL and GC risk and complex allele combinations of these five miRNA SNPs. Additionally, the results of MDR analysis revealed a significant interaction between miR-196a2 rs11614913 and Hp in the risk of GPL. Although the association between these five miRNA SNPs and different cancer risks has been extensively studied, no studies exploring the interactions between them, and between them and Hp infection in GPL as well as GC risk have been published. Our results suggested, to some extent, that miR-196a2 rs11614913 and miR-27a rs895819 may be independent risk factors for GPL or GC risk. And the interaction between miR-196a2 rs11614913 and Hp further affected the susceptibility of GPL.

Some recent studies have identified differences in the expression patterns of some miRNAs between Hp-infected and uninfected normal and cancerous gastric mucosa, suggesting that abnormal miRNA expression may play a key role in the evolution of Hp induced benign to malignant gastric pathological changes (Matsushima et al., 2011; Chang et al., 2015). Up-regulated miR-146a has been shown to promote the apoptosis of Hp-infected human gastric cancer cells by inhibiting COX-2 (Wu et al., 2014). Another study reported that Hp can enhance PGE2 production by inducing COX-2/PGE2 signaling, which leads to the hypermethylated state of miR-149 in cancer-associated fibroblasts (Li et al., 2015). And part of the oncogenic mechanism of miR-27a may be associated with *H. pylori*-induced, Cag-A-dependent G2/M progression in gastric epithelial cells (Peek et al., 1999). Therefore, we detailly explored the differences in the expressions of these five miRNAs among different pathological alterations in HPGD. The results confirmed that expressions of miR-499, miR-196a2 and miR-27a were significantly higher in GC group among Hp-positive subjects. Nevertheless, there were no statistically significant differences in miRNA expressions between pathological groups in Hp-negative subjects. Those indicated that miR-499, miR-196a2 and miR-27a might function as oncogenes in Hp-induced gastric mucosal carcinogenesis. Besides, correlation analysis results revealed that miR-196a2 expression level was positively correlated with the severity of inflammation, inflammatory activity, atrophy, intestinal metaplasia and dysplasia in gastric mucosa. This partly supported our previous hypothesis that miR-196a2 may play an oncogenic role in Hp induced gastric carcinogenesis process. Secondly, there was a positive correlation between expression of miR-146a and the severity of Hp infection and inflammation. This was similar to the findings of Liu et al. (2010) who found that Hp infection can induce the upregulation of miR-146a in an NF-κB-dependent manner. Conversely, miR-149 expression was negatively correlated with the severity of Hp infection, inflammation, inflammatory activity, atrophy, and dysplasia in gastric mucosa. It potentially suggested that miR-149 may play a role as a tumor suppressor gene in the Hp-induced pathological evolution of gastric mucosa. Previous studies have demonstrated that Hp infection can suppress miR-149 expression in human gastric cancer tissues through abnormal DNA methylation (Li et al., 2015). Also, miR-149 has been found to be downregulated in chondrocytes from patients with osteoarthritis and it correlates with increased expression of pro-inflammatory cytokines including IL-1β (Santini et al., 2014). Finally, we also found that miR-146a rs2910164 was associated with its altered expression level. Compared with miR-146a rs2910164 CC genotype, miR-146a expression was higher in CG/GG genotype carriers in NOR, CI and GC group. Jazdzewski et al. (2008) also discovered that rs2910164 of pre-miR-146a affected the expression of mature miR-146a, and the amount of mature miR-146a from C allele was reduced compared to G allele. Based on the above findings, we hypothesized that the combined effects between these five miRNAs and their interactions with Hp infection are jointly involved in the progression of benign to malignant lesions in gastric mucosa and influence the susceptibility to GPL or GC.

However, this study still has some limitations and unsolved issues. First, this study is a single-center study with a small sample size, so there is the possibility of selective bias. Therefore, we need to validate these results in subsequent multicenter and large-scale studies. Second, the detailed molecular biological mechanism of miRNAs’ role in the evolution of Hp-induced gastric mucosal pathology needs to be determined by further studies.

## Conclusion

Our study suggested that miR-196a2 rs11614913 CT genotype and miR-27a rs895819 CT genotype and C allele may be independent risk factors for GPL or GC risk. Meanwhile, the synergistic effects between multiple miRNA SNPs and their interactions with Hp in the pathological evolution of gastric mucosa cannot be ignored. Finally, the upregulation of miR-499, miR-196a2 and miR-27a caused by Hp infection may be an important mechanism of gastric mucosal carcinogenesis and affect the susceptibility of Hp-related gastric diseases.

## Data Availability

The original contributions presented in the study are included in the article/Supplementary Material, further inquiries can be directed to the corresponding author.

## References

[B1] Aziz M. A. Akter T. Islam M. S. (2022). Effect of miR-196a2 rs11614913 polymorphism on cancer susceptibility: Evidence from an updated meta-analysis. Technol. Cancer Res. Treat. 21, 15330338221109798. 10.1177/15330338221109798 35770306 PMC9251994

[B2] Baj J. Forma A. Sitarz M. Portincasa P. Garruti G. Krasowska D. (2021). *Helicobacter pylori* virulence factors-mechanisms of bacterial pathogenicity in the gastric microenvironment. Cells 10 (1), 27. 10.3390/cells10010027 PMC7824444 33375694

[B3] Bansal C. Sharma K. L. Misra S. Srivastava A. N. Mittal B. Singh U. S. (2014). Common genetic variants in pre-microRNAs and risk of breast cancer in the North Indian population. eCancerMedicalScience 8, 473. 10.3332/ecancer.2014.473 25374621 PMC4208924

[B4] Bartel D. P. (2004). MicroRNAs: Genomics, biogenesis, mechanism, and function. Cell 116 (2), 281–297. 10.1016/s0092-8674(04)00045-5 14744438

[B5] Bravo D. Hoare A. Soto C. Valenzuela M. A. Quest A. F. G. (2018). *Helicobacter pylori* in human health and disease: Mechanisms for local gastric and systemic effects. World J. Gastroenterol. 24 (28), 3071–3089. 10.3748/wjg.v24.i28.3071 30065554 PMC6064966

[B6] Chang H. Kim N. Park J. H. Nam R. H. Choi Y. J. Lee H. S. (2015). Different MicroRNA expression levels in gastric cancer depending on *Helicobacter pylori* infection. Gut Liver 9 (2), 188–196. 10.5009/gnl13371 25167801 PMC4351025

[B7] Chen M. Luo F. Yu J. Xiang G. Jiang D. Pu X. (2016). Common functional polymorphism within miR-146a and miR-196a-2 as susceptibility loci for hepatocellular carcinoma: An updated meta-analysis. Meta gene 7, 40–47. 10.1016/j.mgene.2015.11.002 26862480 PMC4707244

[B8] Dixon M. F. Genta R. M. Yardley J. H. Correa P. Batts K. P. Dahms B. B. (1996). Classification and grading of gastritis. The updated Sydney system. International workshop on the histopathology of gastritis, houston 1994. Am. J. Surg. Pathol. 20 (10), 1161–1181. 10.1097/00000478-199610000-00001 8827022

[B9] Early D. S. Ben-Menachem T. Decker G. A. Evans J. A. Fanelli R. D. Fisher D. A. (2012). Appropriate use of GI endoscopy. Gastrointest. Endosc. 75 (6), 1127–1131. 10.1016/j.gie.2012.01.011 22624807

[B10] Jazdzewski K. Murray E. L. Franssila K. Jarzab B. Schoenberg D. R. de la Chapelle A. (2008). Common SNP in pre-miR-146a decreases mature miR expression and predisposes to papillary thyroid carcinoma. Proc. Natl. Acad. Sci. U. S. A. 105 (20), 7269–7274. 10.1073/pnas.0802682105 18474871 PMC2438239

[B11] Kamangar F. Sheikhattari P. Mohebtash M. (2011). *Helicobacter pylori* and its effects on human health and disease. Arch. Iran. Med. 14 (3), 192–199. 21529109

[B12] Kim J. Yum S. Kang C. Kang S.-J. (2016). Gene-gene interactions in gastrointestinal cancer susceptibility. Oncotarget 7 (41), 67612–67625. 10.18632/oncotarget.11701 27588484 PMC5341900

[B13] Li M. Li R. J. Bai H. Xiao P. Liu G. J. Guo Y. W. (2016). Association between the pre-miR-196a2 rs11614913 polymorphism and gastric cancer susceptibility in a Chinese population. Genet. Mol. Res. 15 (2). 10.4238/gmr.15027516 27173281

[B14] Li P. Shan J.-X. Chen X.-H. Zhang D. Su L.-P. Huang X.-Y. (2015). Epigenetic silencing of microRNA-149 in cancer-associated fibroblasts mediates prostaglandin E2/interleukin-6 signaling in the tumor microenvironment. Cell Res. 25 (5), 588–603. 10.1038/cr.2015.51 25916550 PMC4423088

[B15] Liang L. Mai A. Zhou J. Xu E. Wang J. Yang Q. (2022). Association of miR-146a rs2910164 G/C polymorphism with its abnormal expression and risk of gastric cancer. Chin. J. Med. Genet. 39 (3), 286–292. 10.3760/cma.j.cn511374-20200809-00594 35315037

[B16] Liu Z. Xiao B. Tang B. Li B. Li N. Zhu E. (2010). Up-regulated microRNA-146a negatively modulate *Helicobacter pylori*-induced inflammatory response in human gastric epithelial cells. Microbes Infect. 12 (11), 854–863. 10.1016/j.micinf.2010.06.002 20542134

[B17] Livak K. J. Schmittgen T. D. (2001). Analysis of relative gene expression data using real-time quantitative PCR and the 2(-Delta Delta C(T)) Method. Methods 25 (4), 402–408. 10.1006/meth.2001.1262 11846609

[B18] Matsushima K. Isomoto H. Inoue N. Nakayama T. Hayashi T. Nakayama M. (2011). MicroRNA signatures in *Helicobacter pylori*-infected gastric mucosa. Int. J. Cancer 128 (2), 361–370. 10.1002/ijc.25348 20333682

[B19] Min K. T. Kim J. W. Jeon Y. J. Jang M. J. Chong S. Y. Oh D. (2012). Association of the miR-146aC > G, 149C > T, 196a2C > T, and 499A > G polymorphisms with colorectal cancer in the Korean population. Mol. Carcinog. 51, E65–E73. 10.1002/mc.21849 22161766

[B20] Mohammadi A. Khanbabaei H. Zandi F. Ahmadi A. Haftcheshmeh S. M. Johnston T. P. (2022). Curcumin: A therapeutic strategy for targeting the *Helicobacter pylori*-related diseases. Microb. Pathog. 166, 105552. 10.1016/j.micpath.2022.105552 35469998

[B21] Nicoloso M. S. Sun H. Spizzo R. Kim H. Wickramasinghe P. Shimizu M. (2010). Single-nucleotide polymorphisms inside MicroRNA target sites influence tumor susceptibility. Cancer Res. 70 (7), 2789–2798. 10.1158/0008-5472.Can-09-3541 20332227 PMC2853025

[B22] Peek R. M. Blaser M. J. Mays D. J. Forsyth M. H. Cover T. L. Song S. Y. (1999). *Helicobacter pylori* strain-specific genotypes and modulation of the gastric epithelial cell cycle. Cancer Res. 59 (24), 6124–6131. 10626802

[B23] Peng S. Kuang Z. Sheng C. Zhang Y. Xu H. Cheng Q. (2010). Association of MicroRNA-196a-2 gene polymorphism with gastric cancer risk in a Chinese population. Dig. Dis. Sci. 55 (8), 2288–2293. 10.1007/s10620-009-1007-x 19834808

[B24] Rong G. Zhu Y. Tang W. Qiu H. Zhang S. (2021). The correlation of microRNA-499 rs3746444 T > C locus with the susceptibility of gastric cancer: From a case-control study to a meta-analysis. Biosci. Rep. 41 (1), BSR20203461. 10.1042/bsr20203461 33319237 PMC7789807

[B25] Santini P. Politi L. Dalla Vedova P. Scandurra R. d'Abusco A. S. (2014). The inflammatory circuitry of miR-149 as a pathological mechanism in osteoarthritis. Rheumatol. Int. 34 (5), 711–716. 10.1007/s00296-013-2754-8 23595570

[B26] Schork N. J. Fallin D. Lanchbury J. S. (2000). Single nucleotide polymorphisms and the future of genetic epidemiology. Clin. Genet. 58 (4), 250–264. 10.1034/j.1399-0004.2000.580402.x 11076050

[B27] Shankaran Z. S. Walter C. E. J. Prakash N. Ramachandiran K. Doss G. P. C. Johnson T. (2020). Investigating the role of microRNA-27a gene polymorphisms and its interactive effect with risk factors in gastrointestinal cancers. Heliyon 6 (3), e03565. 10.1016/j.heliyon.2020.e03565 32190766 PMC7068059

[B28] Sugano K. Tack J. Kuipers E. J. Graham D. Y. El-Omar E. M. Miura S. (2015). Kyoto global consensus report on *Helicobacter pylori* gastritis. Gut 64 (9), 1353–1367. 10.1136/gutjnl-2015-309252 26187502 PMC4552923

[B29] Venneman K. Huybrechts I. Gunter M. J. Vandendaele L. Herrero R. Van Herck K. (2018). The epidemiology of *Helicobacter pylori* infection in europe and the impact of lifestyle on its natural evolution toward stomach cancer after infection: A systematic review. Helicobacter 23 (3), e12483. 10.1111/hel.12483 29635869

[B30] Wu K. Yang L. Li C. Zhu C.-H. Wang X. Yao Y. (2014). MicroRNA-146a enhances *Helicobacter pylori* induced cell apoptosis in human gastric cancer epithelial cells. Asian pac. J. Cancer Prev. 15 (14), 5583–5586. 10.7314/apjcp.2014.15.14.5583 25081668

[B31] Xu Q. Chen T.-j. He C.-y. Sun L.-p. Liu J.-w. Yuan Y. (2017). MiR-27a rs895819 is involved in increased atrophic gastritis risk, improved gastric cancer prognosis and negative interaction with *Helicobacter pylori* . Sci. Rep. 7, 41307. 10.1038/srep41307 28150722 PMC5288699

[B32] Yang X. Li X. Hao X. Tian W. Zhou B. (2020). Association of miR-27a polymorphism with the risk of digestive system cancers. Pathol. Res. Pract. 216 (10), 153115. 10.1016/j.prp.2020.153115 32853952

[B33] Zhang L. Liu Q. Wang F. (2018). Association between miR-149 gene rs2292832 polymorphism and risk of gastric cancer. Arch. Med. Res. 49 (4), 270–277. 10.1016/j.arcmed.2018.09.012 30274913

[B34] Zlotorynski E. (2019). Insights into the kinetics of microRNA biogenesis and turnover. Nat. Rev. Mol. Cell Biol. 20 (9), 511. 10.1038/s41580-019-0164-9 31366986

[B35] Zou D. Liu C. Zhang Q. Li X. Qin G. Huang Q. (2018). Association between polymorphisms in microRN as and ischemic stroke in an asian population: Evidence based on 6, 083 cases and 7, 248 controls. Clin. Interv. Aging 13, 1709–1726. 10.2147/cia.S174000 30254431 PMC6140750

