# Supplementary material for: Functional gene polymorphisms and expression alteration of selected microRNAs and the risk of various gastric lesions in Helicobacter pylori-related gastric diseases
Source: Front Genet. 2023 Jan 12;13:1097543. doi: 10.3389/fgene.2022.1097543 (PMC9878693; doi:10.3389/fgene.2022.1097543)
Supplement: Supplementary file 1 [file Table1.DOCX]

| **Table S1** **Primer sequences of qPCR** | | |
| --- | --- | --- |
| **Gene** | **Upstream(5’to 3’)** | **Downstream(5’to 3’)** |
| miR-146a | CACTCAGCTGTGAGAACTGAATTC | CTGGTGTCGTGGAGTCG |
| miR-196a2 | CACTCAGCTGTAGGTAGTTTCATG | CTGGTGTCGTGGAGTCG |
| miR-499 | CACTCAGCTACAGACTTGCTG | CTGGTGTCGTGGAGTCG |
| miR-149 | CACTCAGCTTCTGGCTCCGTGT | CTGGTGTCGTGGAGTCG |
| miR-27a | CACTCAGCTAGGGCTTAGCTGCT | CTGGTGTCGTGGAGTCG |
| U6 | CACTCAGCTCACGCAAATTCGTG | CTGGTGTCGTGGAGTCG |

| **Table S2 The effect of miRNA polymorphism on their expression level** | | | | | | | |
| --- | --- | --- | --- | --- | --- | --- | --- |
| **SNPs** | **Genotypes** | **NOR(n=35)** | **CI(n=23)** | **GA(n=11)** | **EPL(n=79)** | **SE(n=10)** | **GC(n=45)** |
| miR-499 rs3746444 | AA | 0.58(0.11, 1.28) | 0.93(0.36, 1.72) | 1.29(0.26, 8.72) | 0.45(0.20, 1.46) | 0.72(0.59, 1.43) | 1.54(0.50, 3.70) |
|  | AG+GG | 1.30(0.50, 1.56) | 1.57(0.11, 4.00) | 1.73(0.07, -) | 1.05(0.24, 2.06) | - | 1.48(0.85, 5.39) |
|  | ***P*** | 0.103 | 0.935 | 0.480 | 0.164 | - | 0.613 |
| miR-149  rs2292832 | TT | 1.02(0.34, 4.08) | 0.21(0.05, 0.54) | 0.23(0.18, 0.81) | 0.40(0.20, 0.89) | 0.30(0.23, 0.33) | 0.64(0.24, 1.05) |
|  | CT+CC | 1.10(0.37, 2.29) | 0.46(0.23, 0.74) | 0.28(0.22, -) | 0.40(0.19, 0.97) | 0.45(0.25, 1.25) | 0.20(0.08, 0.95) |
|  | ***P*** | 0.777 | 0.136 | 0.814 | 0.738 | 0.392 | 0.086 |
| miR-196a2  rs11614913 | CC | 0.35(0.05, 2.06) | 0.33(0.17, 18.65) | - | 0.52(0.11, 2.06) | 0.56(0.14, -) | 20.27(6.41, 81.62) |
|  | CT+TT | 0.37(0.23, 0.68) | 0.26(0.09, 0.68) | 2.19(0.73, 8.62) | 0.61(0.19, 2.19) | 4.84(0.75, 10.19) | 3.71(0.23, 0.68) |
|  | ***P*** | 0.986 | 0.371 | - | 0.697 | 0.117 | 0.117 |
| miR-146a  rs2910164 | CC | 0.69(0.26, 1.40) | 0.20(0.14, 1.12) | 1.66(0.15, -) | 1.64(0.73, 4.22) | 0.25(0.05, -) | 0.40(0.14, 1.26) |
|  | CG+GG | 2.11(0.51, 5.16) | 1.46(0.61, 3.30) | 1.25(0.31, 2.49) | 1.91(0.83, 5.46) | 1.25(1.02, 1.62) | 0.97(0.47, 5.70) |
|  | ***P*** | 0.025 | 0.013 | 0.540 | 0.590 | 0.068 | 0.024 |
| miR-27a  rs895819 | TT | 0.39(0.22, 0.70) | 0.28(0.16, 0.48) | 0.16(0.06, 0.41) | 0.33(0.20, 0.52) | 0.30(0.18, 0.46) | 1.09(0.55, 2.17) |
|  | CT+CC | 0.39(0.12, 0.78) | 0.17(0.14, 0.48) | 0.29(0.09, 0.62) | 0.36(0.20, 0.55) | 0.40(0.17, -) | 0.53(0.10, 2.78) |
|  | ***P*** | 0.959 | 0.508 | 0.751 | 0.672 | 0.732 | 0.255 |
| SNPs: single nucleotide polymorphisms; NOR: relative normal group; CI: chronic inflammation group; GA: gastric atrophy group; EPL: early precancerous lesions group; SE: severe dysplasia group; GC: gastric cancer group  *P* value was calculated by Kruskal-Wallis test (non-parametric) | | | | | | | |
