# Supplementary material for: Functional gene polymorphisms and expression alteration of selected microRNAs and the risk of various gastric lesions in Helicobacter pylori-related gastric diseases
Source: Front Genet. 2023 Jan 12;13:1097543. doi: 10.3389/fgene.2022.1097543 (PMC9878693; doi:10.3389/fgene.2022.1097543)
Supplement: Supplementary file 2 [file Image1.PDF]

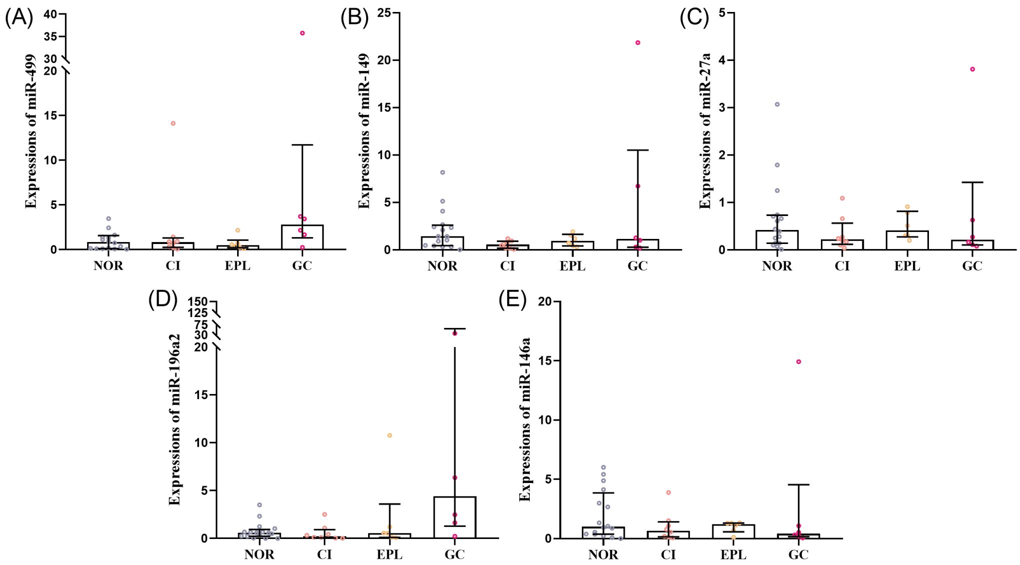

Figure S1. Comparison of miRNA expression levels among different pathological groups in Hp-negative subjects. (A)--(E): comparison of expression levels of miR-499, miR-149, miR-27a, miR-196a2 and miR-146a between different pathological groups. NOR: normal control group; CI: chronic inflammation group; EPL: early precancerous lesion group; GC: gastric cancer group.The column height indicates the median expression level of miRNAs and the error bars indicate interquartile range.
